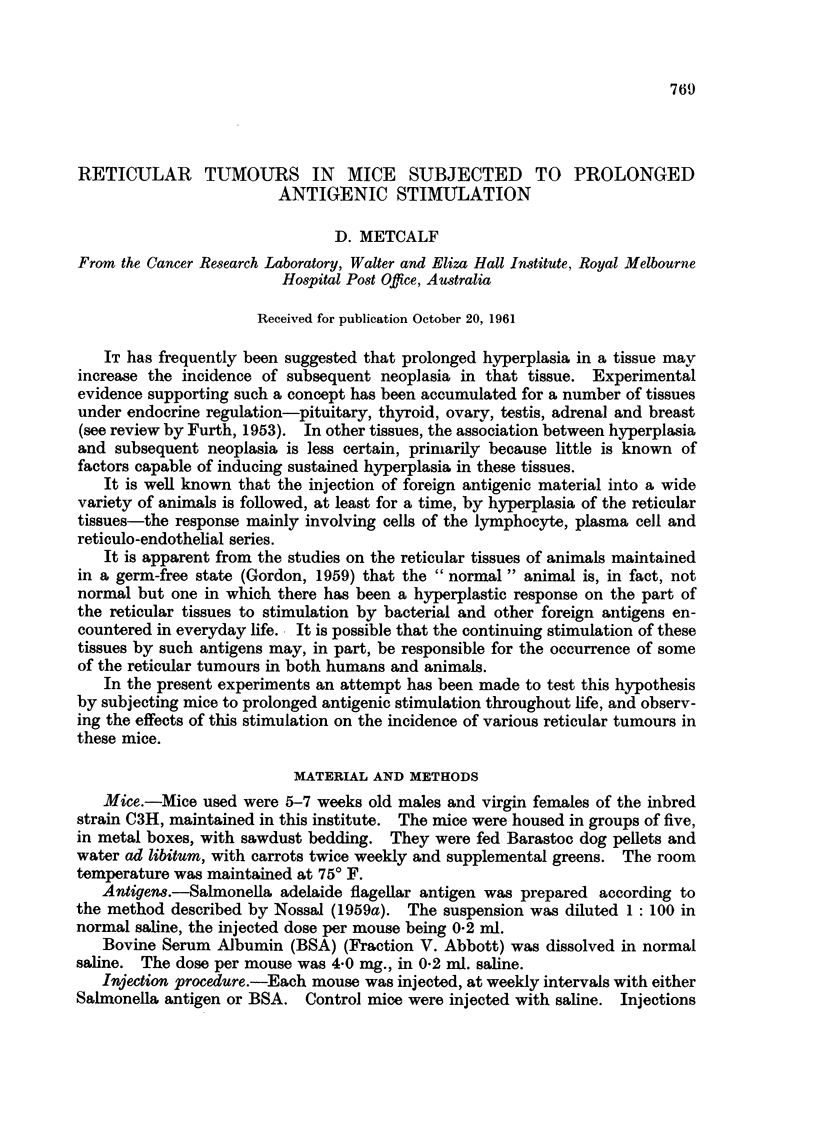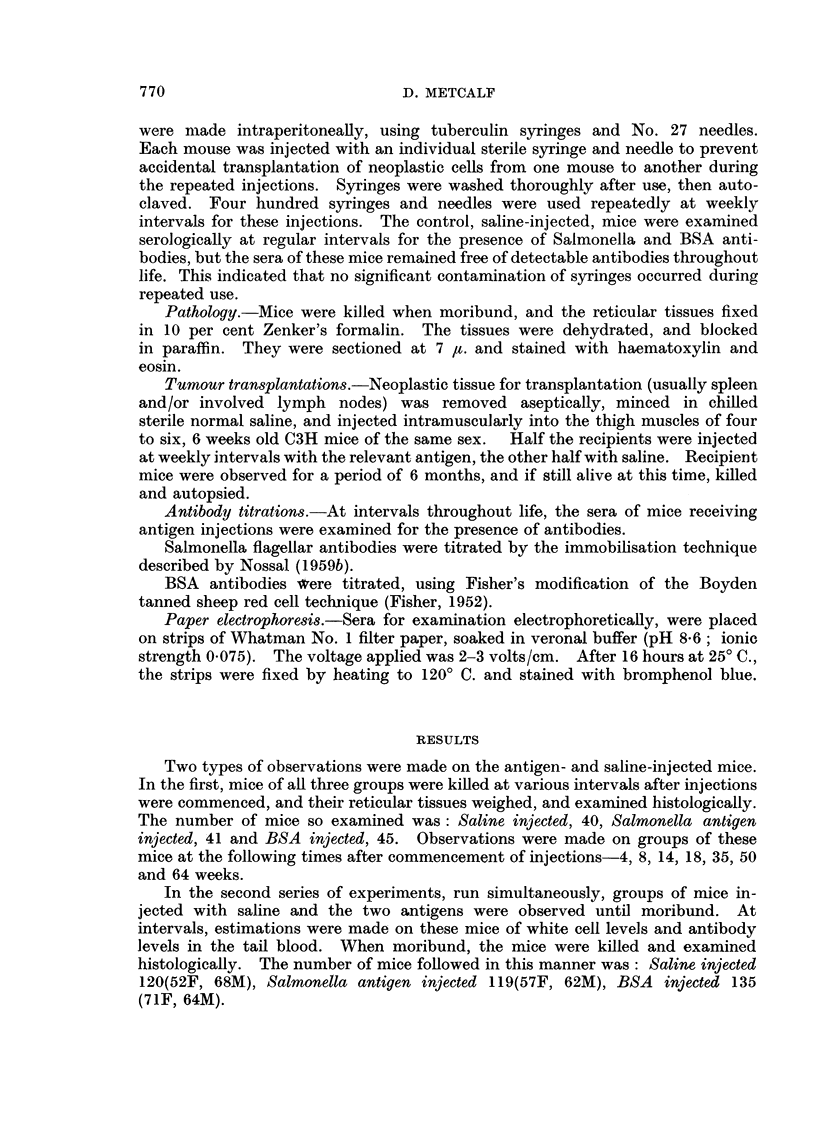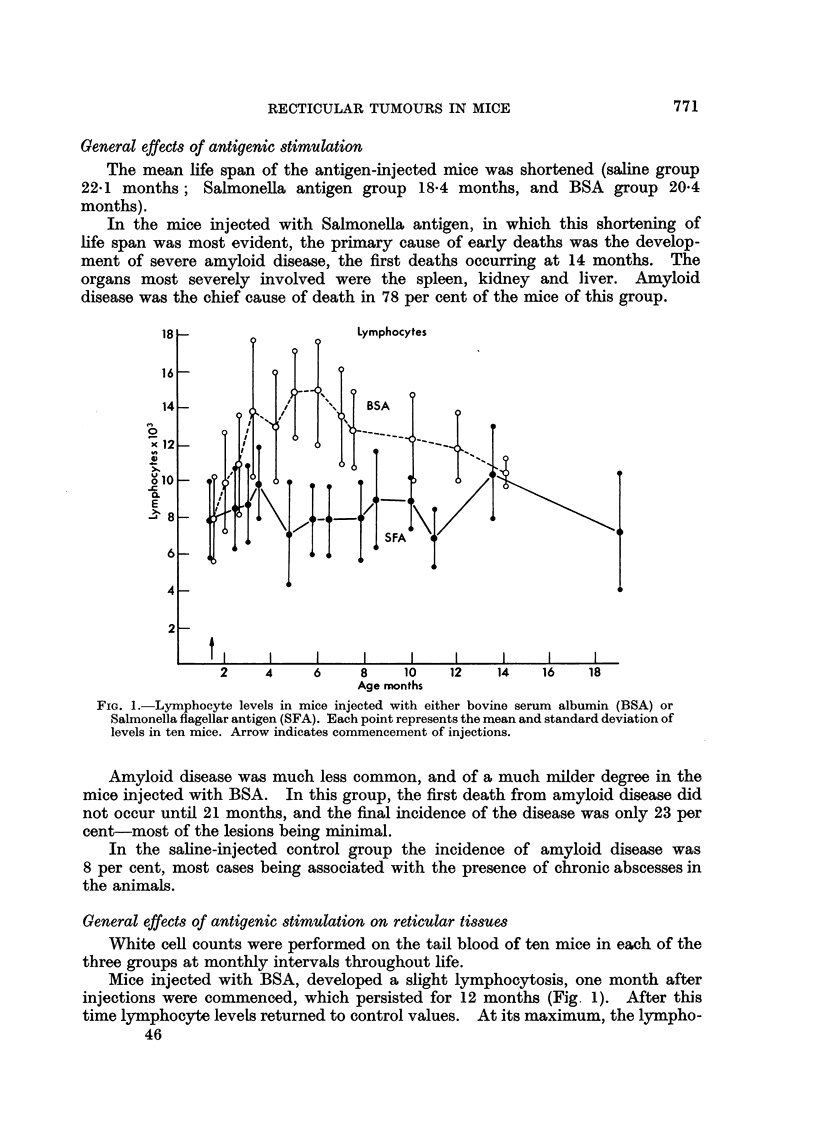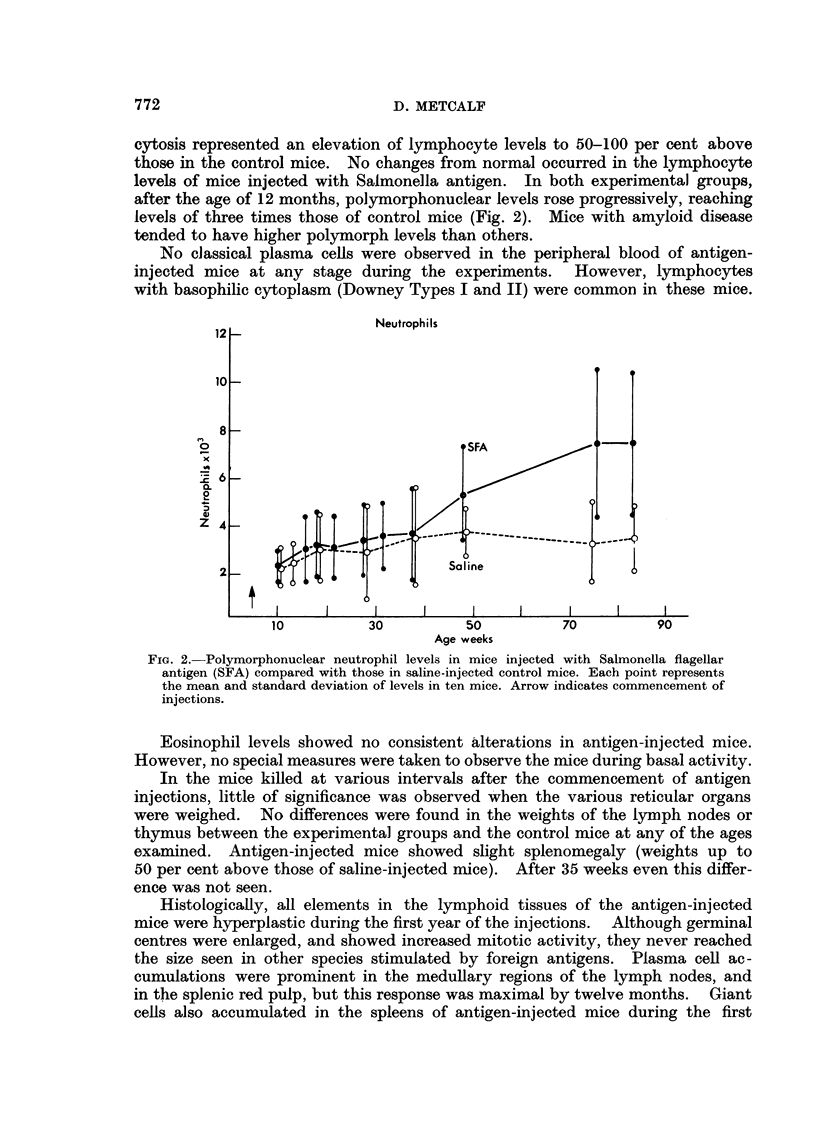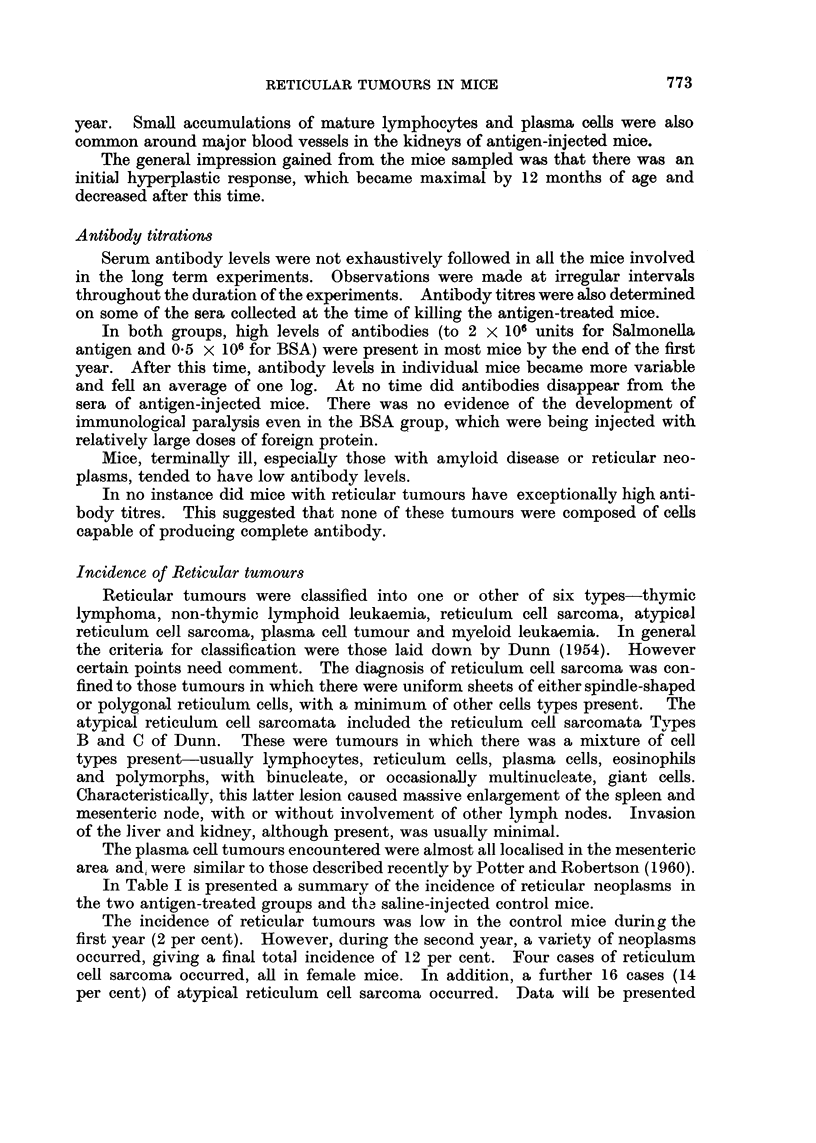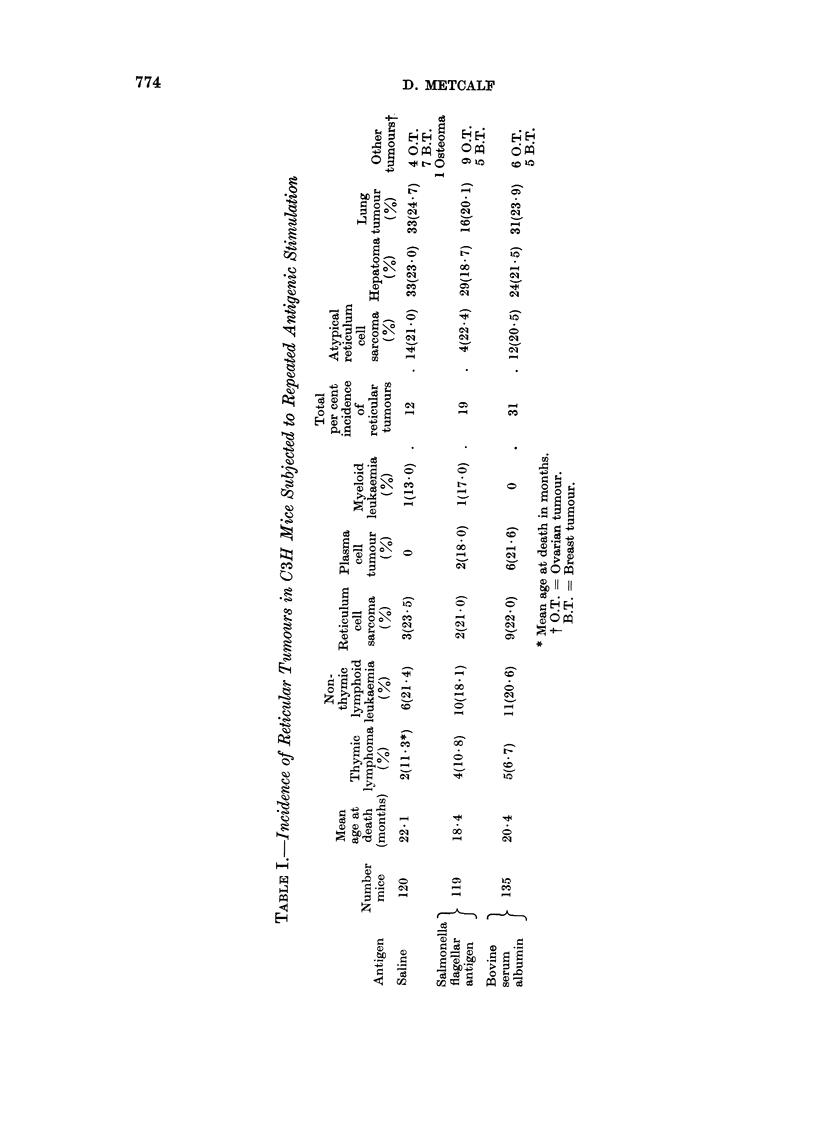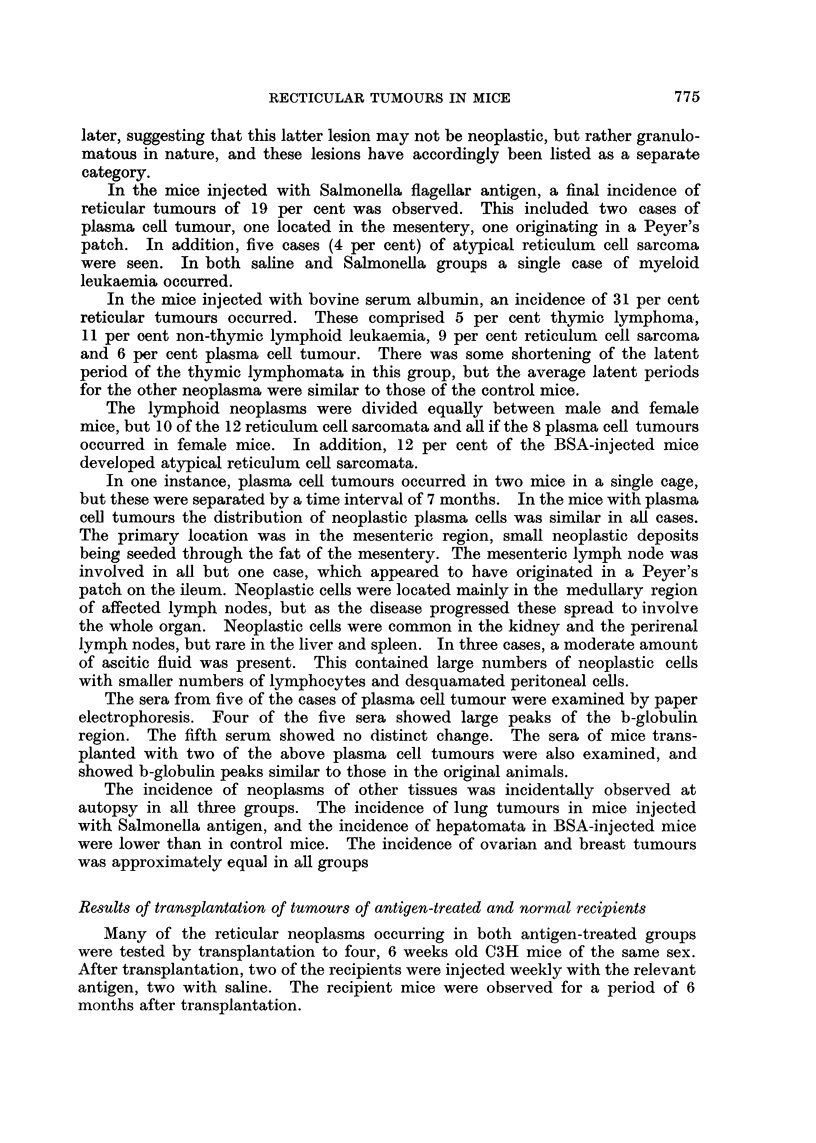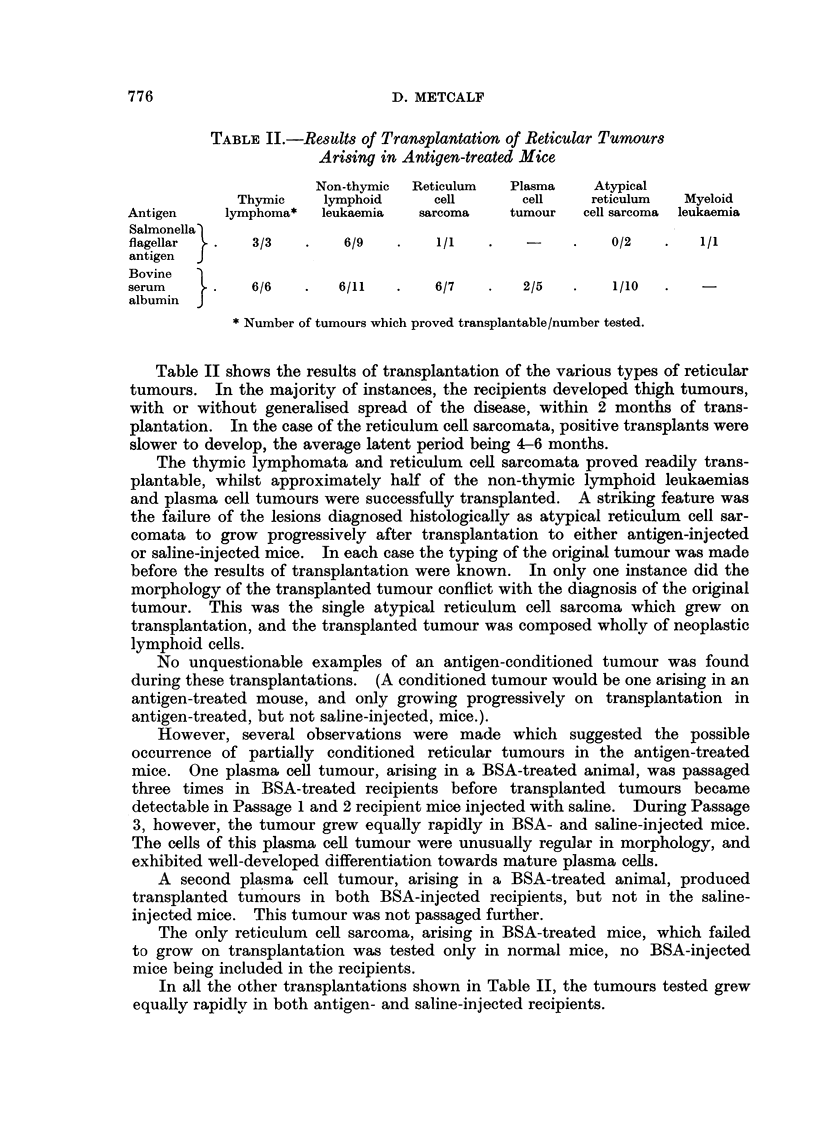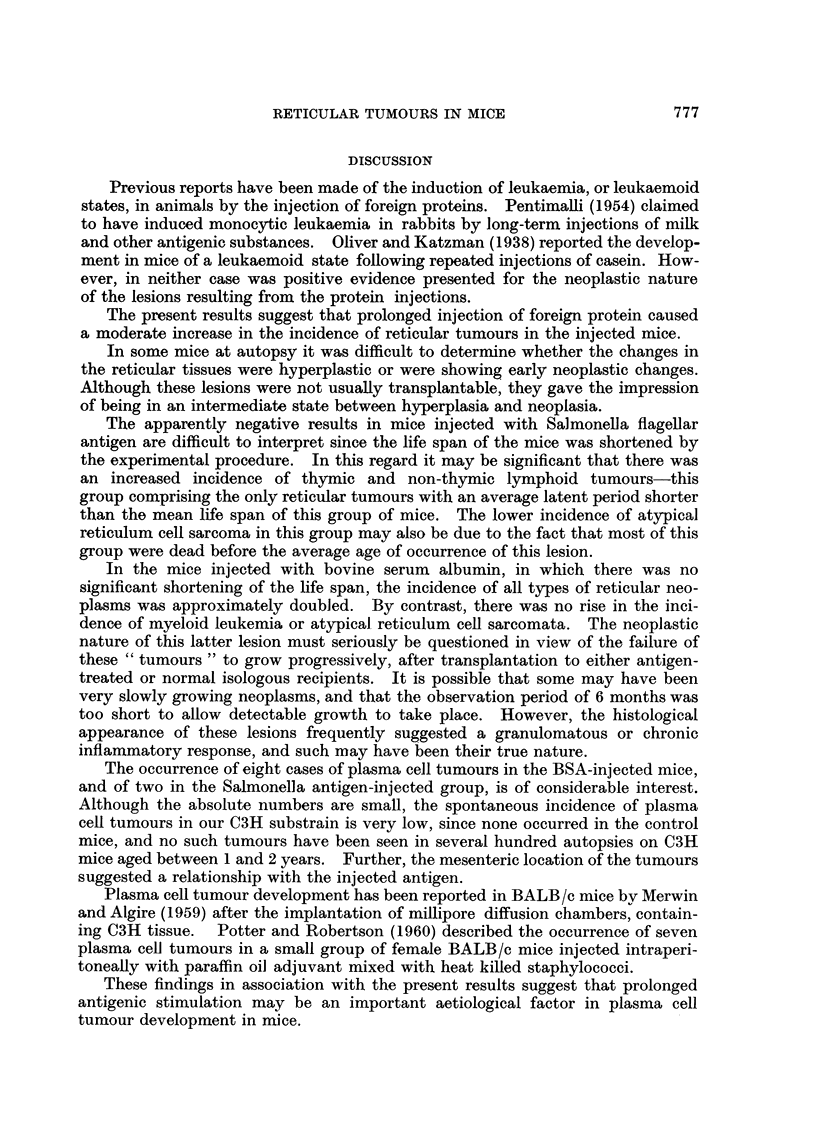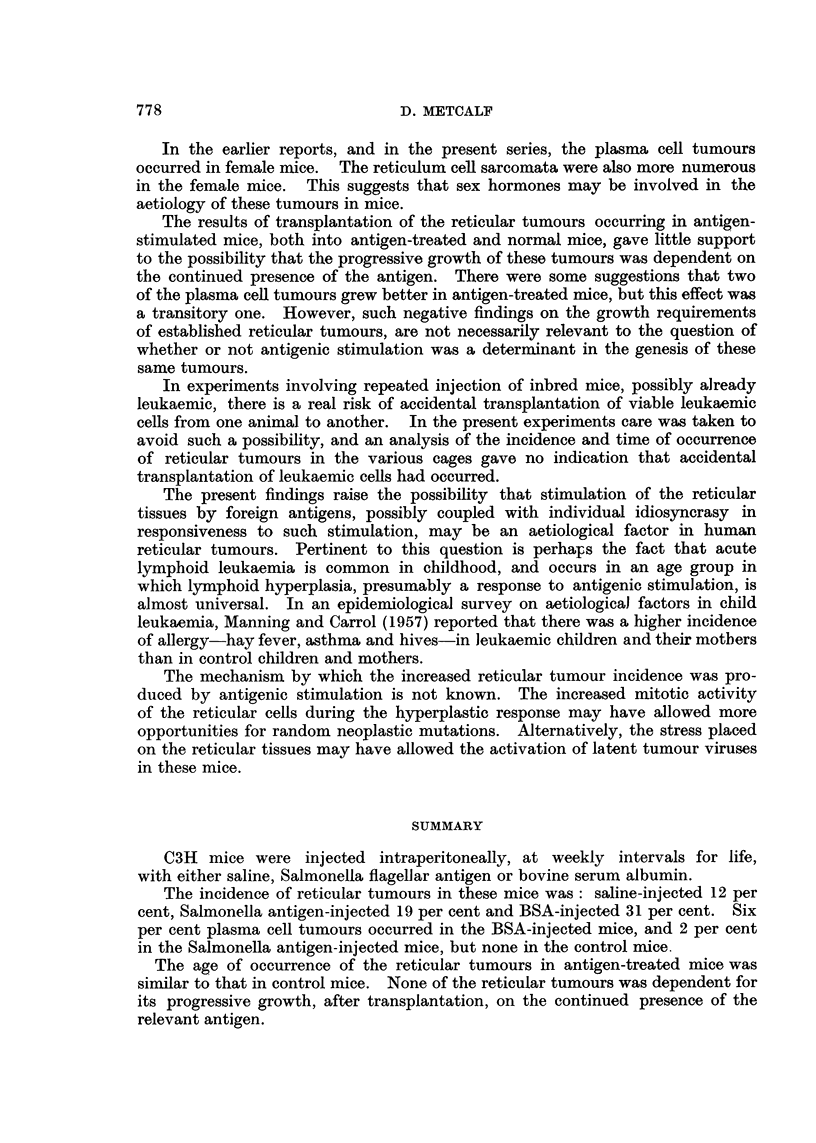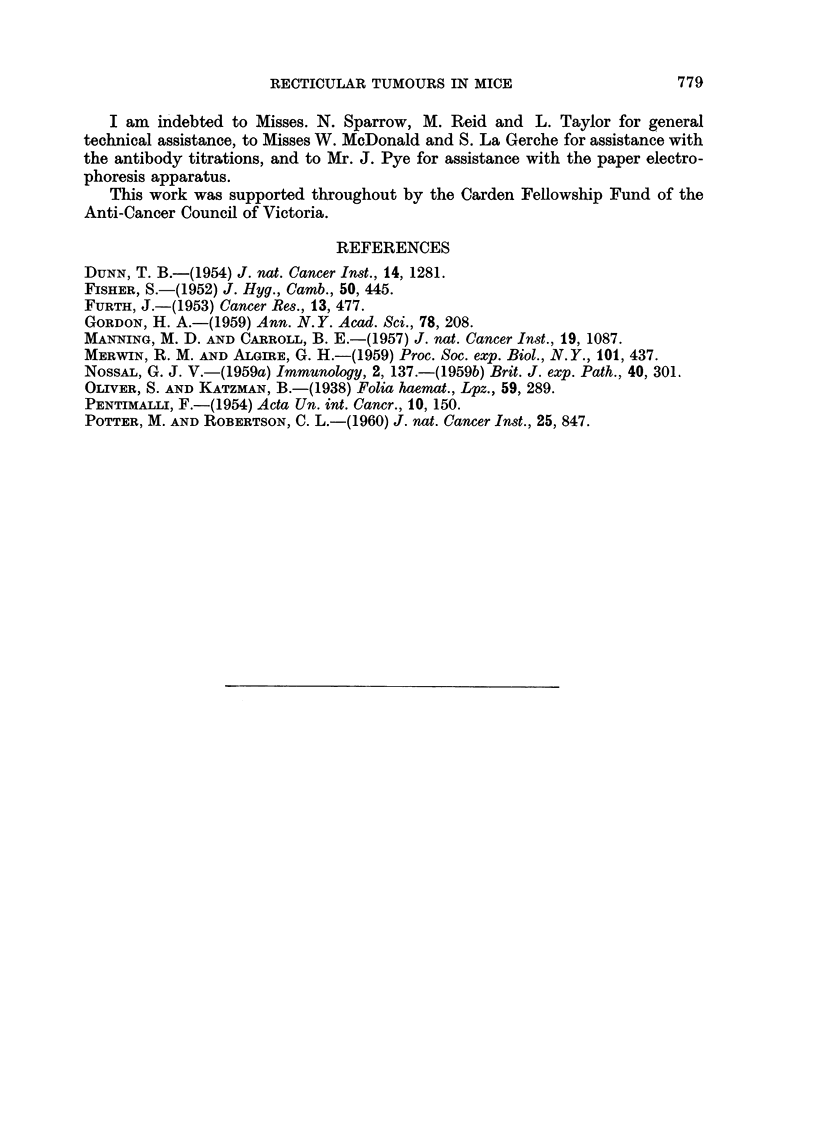# Reticular Tumours in Mice Subjected to Prolonged Antigenic Stimulation

**DOI:** 10.1038/bjc.1961.87

**Published:** 1961-12

**Authors:** D. Metcalf


### RETICULAR TUMOURS IN MICE SUBJECTED TO PROLONGED ANTIGENIC STIMULATION

D. METCALF

*From the Cancer Research Laboratory, Walter and Eliza Hall Institute, Royal Melbourne Hospital Post Office, Australia*

Received for publication October 20, 1961

IT has frequently been suggested that prolonged hyperplasia in a tissue may increase the incidence of subsequent neoplasia in that tissue. Experimental evidence supporting such a concept has been accumulated for a number of tissues under endocrine regulation—pituitary, thyroid, ovary, testis, adrenal and breast (see review by Furth, 1953). In other tissues, the association between hyperplasia and subsequent neoplasia is less certain, primarily because little is known of factors capable of inducing sustained hyperplasia in these tissues.

It is well known that the injection of foreign antigenic material into a wide variety of animals is followed, at least for a time, by hyperplasia of the reticular tissues—the response mainly involving cells of the lymphocyte, plasma cell and reticulo-endothelial series.

It is apparent from the studies on the reticular tissues of animals maintained in a germ-free state (Gordon, 1959) that the " normal " animal is, in fact, not normal but one in which there has been a hyperplastic response on the part of the reticular tissues to stimulation by bacterial and other foreign antigens encountered in everyday life. It is possible that the continuing stimulation of these tissues by such antigens may, in part, be responsible for the occurrence of some of the reticular tumours in both humans and animals.

In the present experiments an attempt has been made to test this hypothesis by subjecting mice to prolonged antigenic stimulation throughout life, and observing the effects of this stimulation on the incidence of various reticular tumours in these mice.

### MATERIAL AND METHODS

*Mice.*—Mice used were 5–7 weeks old males and virgin females of the inbred strain C3H, maintained in this institute. The mice were housed in groups of five, in metal boxes, with sawdust bedding. They were fed Barastoc dog pellets and water *ad libitum*, with carrots twice weekly and supplemental greens. The room temperature was maintained at 75° F.

*Antigens.*—Salmonella adelaide flagellar antigen was prepared according to the method described by Nossal (1959*a*). The suspension was diluted 1 : 100 in normal saline, the injected dose per mouse being 0·2 ml.

Bovine Serum Albumin (BSA) (Fraction V. Abbott) was dissolved in normal saline. The dose per mouse was 4·0 mg., in 0·2 ml. saline.

*Injection procedure.*—Each mouse was injected, at weekly intervals with either Salmonella antigen or BSA. Control mice were injected with saline. Injections were made intraperitoneally, using tuberculin syringes and No. 27 needles. Each mouse was injected with an individual sterile syringe and needle to prevent accidental transplantation of neoplastic cells from one mouse to another during the repeated injections. Syringes were washed thoroughly after use, then autoclaved. Four hundred syringes and needles were used repeatedly at weekly intervals for these injections. The control, saline-injected, mice were examined serologically at regular intervals for the presence of Salmonella and BSA antibodies, but the sera of these mice remained free of detectable antibodies throughout life. This indicated that no significant contamination of syringes occurred during repeated use.

*Pathology.*—Mice were killed when moribund, and the reticular tissues fixed in 10 per cent Zenker's formalin. The tissues were dehydrated, and blocked in paraffin. They were sectioned at 7 $\mu$. and stained with haematoxylin and eosin.

*Tumour transplantations.*—Neoplastic tissue for transplantation (usually spleen and/or involved lymph nodes) was removed aseptically, minced in chilled sterile normal saline, and injected intramuscularly into the thigh muscles of four to six, 6 weeks old C3H mice of the same sex. Half the recipients were injected at weekly intervals with the relevant antigen, the other half with saline. Recipient mice were observed for a period of 6 months, and if still alive at this time, killed and autopsied.

*Antibody titrations.*—At intervals throughout life, the sera of mice receiving antigen injections were examined for the presence of antibodies.

Salmonella flagellar antibodies were titrated by the immobilisation technique described by Nossal (1959*b*).

BSA antibodies were titrated, using Fisher's modification of the Boyden tanned sheep red cell technique (Fisher, 1952).

*Paper electrophoresis.*—Sera for examination electrophoretically, were placed on strips of Whatman No. 1 filter paper, soaked in veronal buffer (pH 8·6 ; ionic strength 0·075). The voltage applied was 2–3 volts/cm. After 16 hours at 25° C., the strips were fixed by heating to 120° C. and stained with bromphenol blue.

### RESULTS

Two types of observations were made on the antigen- and saline-injected mice. In the first, mice of all three groups were killed at various intervals after injections were commenced, and their reticular tissues weighed, and examined histologically. The number of mice so examined was : *Saline injected*, 40, *Salmonella antigen injected*, 41 and *BSA injected*, 45. Observations were made on groups of these mice at the following times after commencement of injections—4, 8, 14, 18, 35, 50 and 64 weeks.

In the second series of experiments, run simultaneously, groups of mice injected with saline and the two antigens were observed until moribund. At intervals, estimations were made on these mice of white cell levels and antibody levels in the tail blood. When moribund, the mice were killed and examined histologically. The number of mice followed in this manner was : *Saline injected* 120(52F, 68M), *Salmonella antigen injected* 119(57F, 62M), *BSA injected* 135 (71F, 64M).

*General effects of antigenic stimulation*

The mean life span of the antigen-injected mice was shortened (saline group 22·1 months ; Salmonella antigen group 18·4 months, and BSA group 20·4 months).

In the mice injected with Salmonella antigen, in which this shortening of life span was most evident, the primary cause of early deaths was the development of severe amyloid disease, the first deaths occurring at 14 months. The organs most severely involved were the spleen, kidney and liver. Amyloid disease was the chief cause of death in 78 per cent of the mice of this group.

FIG. 1.—Lymphocyte levels in mice injected with either bovine serum albumin (BSA) or Salmonella flagellar antigen (SFA). Each point represents the mean and standard deviation of levels in ten mice. Arrow indicates commencement of injections.

Amyloid disease was much less common, and of a much milder degree in the mice injected with BSA. In this group, the first death from amyloid disease did not occur until 21 months, and the final incidence of the disease was only 23 per cent—most of the lesions being minimal.

In the saline-injected control group the incidence of amyloid disease was 8 per cent, most cases being associated with the presence of chronic abscesses in the animals.

*General effects of antigenic stimulation on reticular tissues*

White cell counts were performed on the tail blood of ten mice in each of the three groups at monthly intervals throughout life.

Mice injected with BSA, developed a slight lymphocytosis, one month after injections were commenced, which persisted for 12 months (Fig. 1). After this time lymphocyte levels returned to control values. At its maximum, the lymphocytosis represented an elevation of lymphocyte levels to 50–100 per cent above those in the control mice. No changes from normal occurred in the lymphocyte levels of mice injected with Salmonella antigen. In both experimental groups, after the age of 12 months, polymorphonuclear levels rose progressively, reaching levels of three times those of control mice (Fig. 2). Mice with amyloid disease tended to have higher polymorph levels than others.

No classical plasma cells were observed in the peripheral blood of antigen-injected mice at any stage during the experiments. However, lymphocytes with basophilic cytoplasm (Downey Types I and II) were common in these mice.

FIG. 2.—Polymorphonuclear neutrophil levels in mice injected with Salmonella flagellar antigen (SFA) compared with those in saline-injected control mice. Each point represents the mean and standard deviation of levels in ten mice. Arrow indicates commencement of injections.

Eosinophil levels showed no consistent alterations in antigen-injected mice. However, no special measures were taken to observe the mice during basal activity.

In the mice killed at various intervals after the commencement of antigen injections, little of significance was observed when the various reticular organs were weighed. No differences were found in the weights of the lymph nodes or thymus between the experimental groups and the control mice at any of the ages examined. Antigen-injected mice showed slight splenomegaly (weights up to 50 per cent above those of saline-injected mice). After 35 weeks even this difference was not seen.

Histologically, all elements in the lymphoid tissues of the antigen-injected mice were hyperplastic during the first year of the injections. Although germinal centres were enlarged, and showed increased mitotic activity, they never reached the size seen in other species stimulated by foreign antigens. Plasma cell accumulations were prominent in the medullary regions of the lymph nodes, and in the splenic red pulp, but this response was maximal by twelve months. Giant cells also accumulated in the spleens of antigen-injected mice during the first year. Small accumulations of mature lymphocytes and plasma cells were also common around major blood vessels in the kidneys of antigen-injected mice.

The general impression gained from the mice sampled was that there was an initial hyperplastic response, which became maximal by 12 months of age and decreased after this time.

*Antibody titrations*

Serum antibody levels were not exhaustively followed in all the mice involved in the long term experiments. Observations were made at irregular intervals throughout the duration of the experiments. Antibody titres were also determined on some of the sera collected at the time of killing the antigen-treated mice.

In both groups, high levels of antibodies (to $2 \times 10^6$ units for Salmonella antigen and $0{\cdot}5 \times 10^6$ for BSA) were present in most mice by the end of the first year. After this time, antibody levels in individual mice became more variable and fell an average of one log. At no time did antibodies disappear from the sera of antigen-injected mice. There was no evidence of the development of immunological paralysis even in the BSA group, which were being injected with relatively large doses of foreign protein.

Mice, terminally ill, especially those with amyloid disease or reticular neoplasms, tended to have low antibody levels.

In no instance did mice with reticular tumours have exceptionally high antibody titres. This suggested that none of these tumours were composed of cells capable of producing complete antibody.

*Incidence of Reticular tumours*

Reticular tumours were classified into one or other of six types—thymic lymphoma, non-thymic lymphoid leukaemia, reticulum cell sarcoma, atypical reticulum cell sarcoma, plasma cell tumour and myeloid leukaemia. In general the criteria for classification were those laid down by Dunn (1954). However certain points need comment. The diagnosis of reticulum cell sarcoma was confined to those tumours in which there were uniform sheets of either spindle-shaped or polygonal reticulum cells, with a minimum of other cells types present. The atypical reticulum cell sarcomata included the reticulum cell sarcomata Types B and C of Dunn. These were tumours in which there was a mixture of cell types present—usually lymphocytes, reticulum cells, plasma cells, eosinophils and polymorphs, with binucleate, or occasionally multinucleate, giant cells. Characteristically, this latter lesion caused massive enlargement of the spleen and mesenteric node, with or without involvement of other lymph nodes. Invasion of the liver and kidney, although present, was usually minimal.

The plasma cell tumours encountered were almost all localised in the mesenteric area and were similar to those described recently by Potter and Robertson (1960).

In Table I is presented a summary of the incidence of reticular neoplasms in the two antigen-treated groups and the saline-injected control mice.

The incidence of reticular tumours was low in the control mice during the first year (2 per cent). However, during the second year, a variety of neoplasms occurred, giving a final total incidence of 12 per cent. Four cases of reticulum cell sarcoma occurred, all in female mice. In addition, a further 16 cases (14 per cent) of atypical reticulum cell sarcoma occurred. Data will be presented

TABLE I.—*Incidence of Reticular Tumours in C3H Mice Subjected to Repeated Antigenic Stimulation*

| Antigen | Number mice | Mean age at death (months) | Thymic lymphoma (%) | Non-thymic lymphoid leukaemia (%) | Reticulum cell sarcoma (%) | Plasma cell tumour (%) | Myeloid leukaemia (%) | Total per cent incidence of reticular tumours | Atypical reticulum cell sarcoma (%) | Hepatoma (%) | Lung tumour (%) | Other tumours† |
|---|---|---|---|---|---|---|---|---|---|---|---|---|
| Saline | 120 | 22·1 | 2(11·3*) | 6(21·4) | 3(23·5) | 0 | 1(13·0) | 12 | 14(21·0) | 33(23·0) | 33(24·7) | 4 O.T. 7 B.T. 1 Osteoma |
| Salmonella flagellar antigen | 119 | 18·4 | 4(10·8) | 10(18·1) | 2(21·0) | 2(18·0) | 1(17·0) | 19 | 4(22·4) | 29(18·7) | 16(20·1) | 9 O.T. 5 B.T. |
| Bovine serum albumin | 135 | 20·4 | 5(6·7) | 11(20·6) | 9(22·0) | 6(21·6) | 0 | 31 | 12(20·5) | 24(21·5) | 31(23·9) | 6 O.T. 5 B.T. |

* Mean age at death in months.

† O.T. = Ovarian tumour.

B.T. = Breast tumour.

later, suggesting that this latter lesion may not be neoplastic, but rather granulomatous in nature, and these lesions have accordingly been listed as a separate category.

In the mice injected with Salmonella flagellar antigen, a final incidence of reticular tumours of 19 per cent was observed. This included two cases of plasma cell tumour, one located in the mesentery, one originating in a Peyer's patch. In addition, five cases (4 per cent) of atypical reticulum cell sarcoma were seen. In both saline and Salmonella groups a single case of myeloid leukaemia occurred.

In the mice injected with bovine serum albumin, an incidence of 31 per cent reticular tumours occurred. These comprised 5 per cent thymic lymphoma, 11 per cent non-thymic lymphoid leukaemia, 9 per cent reticulum cell sarcoma and 6 per cent plasma cell tumour. There was some shortening of the latent period of the thymic lymphomata in this group, but the average latent periods for the other neoplasma were similar to those of the control mice.

The lymphoid neoplasms were divided equally between male and female mice, but 10 of the 12 reticulum cell sarcomata and all if the 8 plasma cell tumours occurred in female mice. In addition, 12 per cent of the BSA-injected mice developed atypical reticulum cell sarcomata.

In one instance, plasma cell tumours occurred in two mice in a single cage, but these were separated by a time interval of 7 months. In the mice with plasma cell tumours the distribution of neoplastic plasma cells was similar in all cases. The primary location was in the mesenteric region, small neoplastic deposits being seeded through the fat of the mesentery. The mesenteric lymph node was involved in all but one case, which appeared to have originated in a Peyer's patch on the ileum. Neoplastic cells were located mainly in the medullary region of affected lymph nodes, but as the disease progressed these spread to involve the whole organ. Neoplastic cells were common in the kidney and the perirenal lymph nodes, but rare in the liver and spleen. In three cases, a moderate amount of ascitic fluid was present. This contained large numbers of neoplastic cells with smaller numbers of lymphocytes and desquamated peritoneal cells.

The sera from five of the cases of plasma cell tumour were examined by paper electrophoresis. Four of the five sera showed large peaks of the b-globulin region. The fifth serum showed no distinct change. The sera of mice transplanted with two of the above plasma cell tumours were also examined, and showed b-globulin peaks similar to those in the original animals.

The incidence of neoplasms of other tissues was incidentally observed at autopsy in all three groups. The incidence of lung tumours in mice injected with Salmonella antigen, and the incidence of hepatomata in BSA-injected mice were lower than in control mice. The incidence of ovarian and breast tumours was approximately equal in all groups

*Results of transplantation of tumours of antigen-treated and normal recipients*

Many of the reticular neoplasms occurring in both antigen-treated groups were tested by transplantation to four, 6 weeks old C3H mice of the same sex. After transplantation, two of the recipients were injected weekly with the relevant antigen, two with saline. The recipient mice were observed for a period of 6 months after transplantation.

TABLE II.—*Results of Transplantation of Reticular Tumours Arising in Antigen-treated Mice*

| Antigen | Thymic lymphoma* | Non-thymic lymphoid leukaemia | Reticulum cell sarcoma | Plasma cell tumour | Atypical reticulum cell sarcoma | Myeloid leukaemia |
|---|---|---|---|---|---|---|
| Salmonella flagellar antigen | 3/3 | 6/9 | 1/1 | — | 0/2 | 1/1 |
| Bovine serum albumin | 6/6 | 6/11 | 6/7 | 2/5 | 1/10 | — |

* Number of tumours which proved transplantable/number tested.

Table II shows the results of transplantation of the various types of reticular tumours. In the majority of instances, the recipients developed thigh tumours, with or without generalised spread of the disease, within 2 months of transplantation. In the case of the reticulum cell sarcomata, positive transplants were slower to develop, the average latent period being 4–6 months.

The thymic lymphomata and reticulum cell sarcomata proved readily transplantable, whilst approximately half of the non-thymic lymphoid leukaemias and plasma cell tumours were successfully transplanted. A striking feature was the failure of the lesions diagnosed histologically as atypical reticulum cell sarcomata to grow progressively after transplantation to either antigen-injected or saline-injected mice. In each case the typing of the original tumour was made before the results of transplantation were known. In only one instance did the morphology of the transplanted tumour conflict with the diagnosis of the original tumour. This was the single atypical reticulum cell sarcoma which grew on transplantation, and the transplanted tumour was composed wholly of neoplastic lymphoid cells.

No unquestionable examples of an antigen-conditioned tumour was found during these transplantations. (A conditioned tumour would be one arising in an antigen-treated mouse, and only growing progressively on transplantation in antigen-treated, but not saline-injected, mice.).

However, several observations were made which suggested the possible occurrence of partially conditioned reticular tumours in the antigen-treated mice. One plasma cell tumour, arising in a BSA-treated animal, was passaged three times in BSA-treated recipients before transplanted tumours became detectable in Passage 1 and 2 recipient mice injected with saline. During Passage 3, however, the tumour grew equally rapidly in BSA- and saline-injected mice. The cells of this plasma cell tumour were unusually regular in morphology, and exhibited well-developed differentiation towards mature plasma cells.

A second plasma cell tumour, arising in a BSA-treated animal, produced transplanted tumours in both BSA-injected recipients, but not in the saline-injected mice. This tumour was not passaged further.

The only reticulum cell sarcoma, arising in BSA-treated mice, which failed to grow on transplantation was tested only in normal mice, no BSA-injected mice being included in the recipients.

In all the other transplantations shown in Table II, the tumours tested grew equally rapidly in both antigen- and saline-injected recipients.

### DISCUSSION

Previous reports have been made of the induction of leukaemia, or leukaemoid states, in animals by the injection of foreign proteins. Pentimalli (1954) claimed to have induced monocytic leukaemia in rabbits by long-term injections of milk and other antigenic substances. Oliver and Katzman (1938) reported the development in mice of a leukaemoid state following repeated injections of casein. However, in neither case was positive evidence presented for the neoplastic nature of the lesions resulting from the protein injections.

The present results suggest that prolonged injection of foreign protein caused a moderate increase in the incidence of reticular tumours in the injected mice.

In some mice at autopsy it was difficult to determine whether the changes in the reticular tissues were hyperplastic or were showing early neoplastic changes. Although these lesions were not usually transplantable, they gave the impression of being in an intermediate state between hyperplasia and neoplasia.

The apparently negative results in mice injected with Salmonella flagellar antigen are difficult to interpret since the life span of the mice was shortened by the experimental procedure. In this regard it may be significant that there was an increased incidence of thymic and non-thymic lymphoid tumours—this group comprising the only reticular tumours with an average latent period shorter than the mean life span of this group of mice. The lower incidence of atypical reticulum cell sarcoma in this group may also be due to the fact that most of this group were dead before the average age of occurrence of this lesion.

In the mice injected with bovine serum albumin, in which there was no significant shortening of the life span, the incidence of all types of reticular neoplasms was approximately doubled. By contrast, there was no rise in the incidence of myeloid leukemia or atypical reticulum cell sarcomata. The neoplastic nature of this latter lesion must seriously be questioned in view of the failure of these " tumours " to grow progressively, after transplantation to either antigen-treated or normal isologous recipients. It is possible that some may have been very slowly growing neoplasms, and that the observation period of 6 months was too short to allow detectable growth to take place. However, the histological appearance of these lesions frequently suggested a granulomatous or chronic inflammatory response, and such may have been their true nature.

The occurrence of eight cases of plasma cell tumours in the BSA-injected mice, and of two in the Salmonella antigen-injected group, is of considerable interest. Although the absolute numbers are small, the spontaneous incidence of plasma cell tumours in our C3H substrain is very low, since none occurred in the control mice, and no such tumours have been seen in several hundred autopsies on C3H mice aged between 1 and 2 years. Further, the mesenteric location of the tumours suggested a relationship with the injected antigen.

Plasma cell tumour development has been reported in BALB/c mice by Merwin and Algire (1959) after the implantation of millipore diffusion chambers, containing C3H tissue. Potter and Robertson (1960) described the occurrence of seven plasma cell tumours in a small group of female BALB/c mice injected intraperitoneally with paraffin oil adjuvant mixed with heat killed staphylococci.

These findings in association with the present results suggest that prolonged antigenic stimulation may be an important aetiological factor in plasma cell tumour development in mice.

In the earlier reports, and in the present series, the plasma cell tumours occurred in female mice. The reticulum cell sarcomata were also more numerous in the female mice. This suggests that sex hormones may be involved in the aetiology of these tumours in mice.

The results of transplantation of the reticular tumours occurring in antigen-stimulated mice, both into antigen-treated and normal mice, gave little support to the possibility that the progressive growth of these tumours was dependent on the continued presence of the antigen. There were some suggestions that two of the plasma cell tumours grew better in antigen-treated mice, but this effect was a transitory one. However, such negative findings on the growth requirements of established reticular tumours, are not necessarily relevant to the question of whether or not antigenic stimulation was a determinant in the genesis of these same tumours.

In experiments involving repeated injection of inbred mice, possibly already leukaemic, there is a real risk of accidental transplantation of viable leukaemic cells from one animal to another. In the present experiments care was taken to avoid such a possibility, and an analysis of the incidence and time of occurrence of reticular tumours in the various cages gave no indication that accidental transplantation of leukaemic cells had occurred.

The present findings raise the possibility that stimulation of the reticular tissues by foreign antigens, possibly coupled with individual idiosyncrasy in responsiveness to such stimulation, may be an aetiological factor in human reticular tumours. Pertinent to this question is perhaps the fact that acute lymphoid leukaemia is common in childhood, and occurs in an age group in which lymphoid hyperplasia, presumably a response to antigenic stimulation, is almost universal. In an epidemiological survey on aetiological factors in child leukaemia, Manning and Carrol (1957) reported that there was a higher incidence of allergy—hay fever, asthma and hives—in leukaemic children and their mothers than in control children and mothers.

The mechanism by which the increased reticular tumour incidence was produced by antigenic stimulation is not known. The increased mitotic activity of the reticular cells during the hyperplastic response may have allowed more opportunities for random neoplastic mutations. Alternatively, the stress placed on the reticular tissues may have allowed the activation of latent tumour viruses in these mice.

### SUMMARY

C3H mice were injected intraperitoneally, at weekly intervals for life, with either saline, Salmonella flagellar antigen or bovine serum albumin.

The incidence of reticular tumours in these mice was : saline-injected 12 per cent, Salmonella antigen-injected 19 per cent and BSA-injected 31 per cent. Six per cent plasma cell tumours occurred in the BSA-injected mice, and 2 per cent in the Salmonella antigen-injected mice, but none in the control mice.

The age of occurrence of the reticular tumours in antigen-treated mice was similar to that in control mice. None of the reticular tumours was dependent for its progressive growth, after transplantation, on the continued presence of the relevant antigen.

I am indebted to Misses. N. Sparrow, M. Reid and L. Taylor for general technical assistance, to Misses W. McDonald and S. La Gerche for assistance with the antibody titrations, and to Mr. J. Pye for assistance with the paper electrophoresis apparatus.

This work was supported throughout by the Carden Fellowship Fund of the Anti-Cancer Council of Victoria.

### REFERENCES

DUNN, T. B.—(1954) *J. nat. Cancer Inst.*, **14**, 1281.
FISHER, S.—(1952) *J. Hyg., Camb.*, **50**, 445.
FURTH, J.—(1953) *Cancer Res.*, **13**, 477.
GORDON, H. A.—(1959) *Ann. N.Y. Acad. Sci.*, **78**, 208.
MANNING, M. D. AND CARROLL, B. E.—(1957) *J. nat. Cancer Inst.*, **19**, 1087.
MERWIN, R. M. AND ALGIRE, G. H.—(1959) *Proc. Soc. exp. Biol., N.Y.*, **101**, 437.
NOSSAL, G. J. V.—(1959*a*) *Immunology*, **2**, 137.—(1959*b*) *Brit. J. exp. Path.*, **40**, 301.
OLIVER, S. AND KATZMAN, B.—(1938) *Folia haemat., Lpz.*, **59**, 289.
PENTIMALLI, F.—(1954) *Acta Un. int. Cancr.*, **10**, 150.
POTTER, M. AND ROBERTSON, C. L.—(1960) *J. nat. Cancer Inst.*, **25**, 847.